# Plasmonic Perfect Absorber Utilizing Polyhexamethylene Biguanide Polymer for Carbon Dioxide Gas Sensing Application

**DOI:** 10.3390/ma16072629

**Published:** 2023-03-26

**Authors:** Muhammad Irfan, Yousuf Khan, Atiq Ur Rehman, Naqeeb Ullah, Svetlana N. Khonina, Nikolay L. Kazanskiy, Muhammad A. Butt

**Affiliations:** 1 Nanophotonics Research Group, Department of Electronic Engineering, Balochistan University of Information Technology, Engineering and Management Sciences, Quetta 87300, Pakistan; 2 Samara National Research University, 443086 Samara, Russia; 3 IPSI RAS-Branch of the FSRC “Crystallography and Photonics” RAS, 443001 Samara, Russia; 4 Institute of Microelectronics and Optoelectronics, Warsaw University of Technology, Koszykowa 75, 00-662 Warszawa, Poland

**Keywords:** polyhexamethylene biguanide polymer, perfect absorber, plasmonic gas sensor, carbon dioxide sensor, lithium niobate-based sensing

## Abstract

In this paper a perfect absorber with a photonic crystal cavity (PhC-cavity) is numerically investigated for carbon dioxide (CO_2_) gas sensing application. Metallic structures in the form of silver are introduced for harnessing plasmonic effects to achieve perfect absorption. The sensor comprises a PhC-cavity, silver (Ag) stripes, and a host functional material—Polyhexamethylene biguanide polymer—deposited on the surface of the sensor. The PhC-cavity is implemented within the middle of the cell, helping to penetrate the EM waves into the sublayers of the structure. Therefore, corresponding to the concentration of the CO_2_ gas, as it increases, the refractive index of the host material decreases, causing a blue shift in the resonant wavelength and vice versa of the device. The sensor is used for the detection of 0–524 parts per million (ppm) concentration of the CO_2_ gas, with a maximum sensitivity of 17.32 pm (pico meter)/ppm achieved for a concentration of 366 ppm with a figure of merit (FOM) of 2.9 RIU^−1^. The four-layer device presents a straightforward and compact design that can be adopted in various sensing applications by using suitable host functional materials.

## 1. Introduction

Recent development in the field of photonics and optical technology has opened new frontiers for researchers to explore their usage in various fields, such as integrated components [1], biomedical devices [2], sensing applications [3], industry-based 4.0 era [4], and many more [5]. Similarly, the burning issue of global warming is also being addressed using photonics technology in terms of design and the investigation of different photonics sensors to detect and calculate the number of different gases in the environment [6]. Regarding global warming, CO_2_ is of utmost importance [7] and is the main constituent responsible for climate change; therefore, necessary steps are required to tackle it efficiently [8]. The correct measurement of CO_2_ is an important factor as it has already passed the threshold value of 350 parts per million (ppm) for unavoidable climate change, and is presently prevailing at 420 ppm [9]. For this purpose, several methods/devices, i.e., phase-sensitive [10], optical fibers [11], pyro-phototronic [12], and refractive index [13] are continuously included in the designing of CO_2_-based sensors. Similarly, photonic crystals (PhCs) are also one of the most significant contenders serving as nanostructures that are capable of controlling light at wavelength scales [14]. PhCs are found in nature [15] and can be designed and fabricated artificially as per the specification of the device, i.e., optical sensors [16], nanowires [17], DBRs [18], waveguides [19], and optical filters [20]. However, recently, the localized surface plasmon polariton (LSPP) has gained the attention of researchers both in the field of academia and industry due to their nature of high sensitivity [21] and simplified materials usage [22]. LSPP-based devices require metal layers, i.e., gold (Au) or silver (Ag), for their integration within optical sensors as they possess unique geometrical shapes for this purpose [23]. As an acknowledgeable fact, the high dependency of the surface plasmon resonances (SPR) on the perimeter of the sensors, geometrical shapes, and refractive index of the environment make them ideal candidates for sensing applications utilizing the properties of perfect absorbers.

Perfect absorbers are artificially created structures made of modified properties to satisfy certain conditions of a device in terms of impedance matching, reflection, transmission, and absorbance [24]. It comprises a periodic top layer allowing no reflection of electromagnetic (EM) waves, followed by a layer to dissipate the EM wave and block its transmission from the structure, respectively [25,26]. Moreover, it is shown through research work performed in the areas of perfect absorbers that the material may or may not always comprise MIM sequence as well, as studied in [27], using a dielectric material and a vertical double-pillars sequence of meta-molecules covered by a layer of Au. Therefore, single materials with designing techniques can also harness useful properties, as proven in [28], using a mechanism of degenerate critical coupling (DCC) and quadrupole modes, which have thus led to the creation of diverse applications in the field of sensing, such as plasmonics-based sensors [29], refractive index-based sensors [30], and temperature sensors, all by employing the phenomena of enhanced-optical utilization [31], time-reversed lasers [32], and thin-layered structures [33].

In the literature, numerous sensing devices have been reported to efficiently measure the concentration of CO_2_ gas [34] using functional layers [35] in terms of poly-hexamethylene biguanide (PHMB), which mostly produce a blue shift in the refractive index [36]. An experimental study in [37] uses a functional layer to detect and sense different concentrations of CO_2_ gas in the range of 0–700 ppm using a Fabry–Perot cavity with a maximum achieved sensitivity of 12.2 pm/ppm. To detect and sense different concentrations of CO_2_ and hydrogen (H_2_) gases, the concept of silicon photonic-dual-gas sensor based on wavelength-multiplexed micro-ring resonator array is given in [38], where two different functional layers, i.e., palladium for H_2_ gas and PHMB for CO_2_, are used. Correspondingly, experimental research using a functional layer-coated microbubble resonator is investigated in [39], where a tapered fiber used to measure CO_2_ within a concentration range of 200–700 ppm results in a sensitivity of 0.46 pm/ppm. A design approach in [40] uses a metal-insulator-metal (MIM) waveguide-based structure coupled to a square-ring cavity filled with PHMB functional material to sense the CO_2_ for a concentration range of 0–524 ppm. Concepts of dual-band CO_2_ gas sensor is employed in [41], using a metasurface of metal-TiO_2_-metal covered with a functional material as a sensing polymer yielding sensitivities around 1040 and 1330 nm/ppm for a range of 200–600 ppm of concentration. A design of a CO_2_ gas sensor based on silicon micro-ring refractometric is investigated in [42], using a coating of the functional layer to sense different concentrations, i.e., 0–500 ppm of the CO_2_ gas, attaining a sensitivity value of 6 × 10^−9^ RIU/ppm. To design an efficient CO_2_ gas sensor, [43] uses a graphene layer on a nano-wall honeycomb structure with distributed plasmonics particles, achieving an overall sensitivity of 874 nm/RIU. Moreover, silicon photonics is gaining attention over silicon electronics in terms of speed, power, and bandwidth, specifically relating to the gas sensing applications as studied in [44], which investigates different techniques and design approaches, such as the PhC-based gas sensor, dual gas sensors, and trace gas sensors. Apart from these, using the long period gratings (LPG) coupled to a single mode, propagation in the silicon layer is explored in [45] for the efficient detection of the different concentrations of CO_2_ gas using a functional polymer layer of PHMB. Similarly, on-chip optical sensors have vast applications in the field of gas-sensing applications; a detailed study on different perspectives of this topic is investigated in [46], using group-IV materials for the purpose.

This research study presents a simple and compact design of the CO_2_ gas sensor, comprising multi-structured layers of different materials providing high sensitivity and tunability in the infrared-A region (IR-A) around 940–960 nm. The multi-structured layer comprises a perfect electric conductor (PEC), silicon dioxide (SiO_2_), lithium niobate (LiNbO_3_), silver (Ag) stripes, and PHMB functional layer, resulting in the perimeters of the sensor in (x and y) directions as being 1000 nm each with a height of 240 nm, including the PHMB functional layer. Moreover, Ag stripes are used to achieve the plasmonic characteristics of the sensor and act as active metal layers that help configure EM energy in producing plasmons on the boundary of metal-polymer and metal-dielectric layers. Therefore, the Ag stripes in combination with the Ag ring are enhancing this effect. Other alternative materials can be Au, aluminum (Al), copper (Cu), and Ag. However, gold and silver are the most widely used plasmonic materials for their non-corrosive effects and lesser metal-induced losses. For the foundation base, LiNbO_3_ is used in this research, acting as a better absorbing material in the case of plasmonics-based sensors [47] as compared to the traditional dielectric materials, i.e., MgF_2_ and TiO_2_.

## 2. Sensor Design and Materials

The sensor is designed while keeping in mind the targets to keep the design compact and at the same time achieve a good level of sensitivity. Therefore, the foundation base of the sensor is made up of the LiNbO_3_ deposited on a layer of SiO_2_, which is a new entry in the optical materials, having better electro-optic, piezoelectric, and nonlinear optical characteristics [48] and is therefore limited to a thickness of 100 nm. Moreover, it also helps in a better confinement and suppression of the energy within the structure when the sensitivity of the IR-absorbers-based devices increases. Due to these factors, it is considered a better choice in laser-based and other optoelectronic devices [49]. Similarly, for plasmonics characteristics, Ag stripes are carved with cross-geometry on the LiNbO_3_ layer having a height and width of 1 nm and 60 nm, respectively.

**Figure 1:**
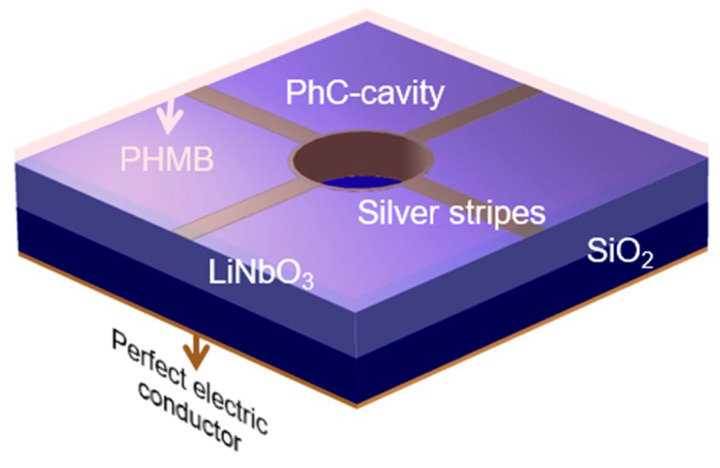
Design of the CO_2_ gas sensor, showing the layout of different layers.

For sensing the CO_2_ gas, a functional layer of PHMB is deposited over the top of LiNbO_3_, which is a member of the guanidine polymer family and is known for capturing the molecules of CO_2_ by sensing its different concentrations at room temperature and normal atmospheric pressure [50]. Moreover, it is used due to its ability to vary its refractive index in response to an ambient change in the concentration of CO_2_ gas in the environment, as well as due to its unique property of effectively sensing CO_2_ gas without the need for water vapor as a catalyst to make CO_2_ molecules heavier for better sensitivity calculations [51]. Moreover, during sensing, CO_2_ molecules are absorbed within the layer of the PHMB, due to which, its refractive index and sensitivity change, and as a result produce a blue shift in the resonant wavelength in the output spectrum. As the simulation data signifies, a ring of Ag is also inserted alongside Ag stripes into the PhC-cavity for stronger and more prominent plasmonic effects. The outer and inner radii of this ring are selected as 150 nm and 140 nm, respectively, along with a thickness of 10 nm. The perfect absorber sensor model is shown in Figure 1.

**Figure 2:**
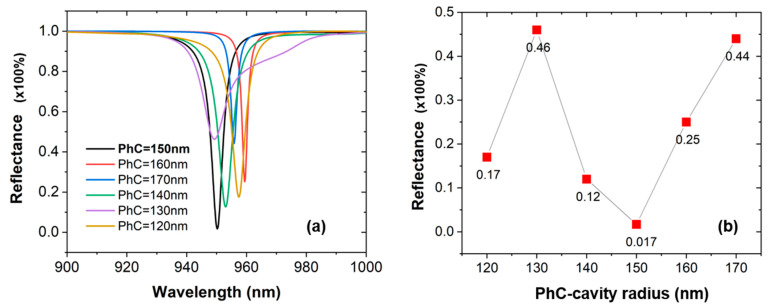
(**a**) Values of the radii of the PhC-cavity for the optimization of the sensor. (**b**) Output spectra showing a drop in the reflectance of the sensor with respect to the change in the PhC-cavity radius.

For a better penetration and absorbance of the electromagnetic (EM) waves within the sub-layers of the design, a PhC-cavity is implemented within the middle of the structure. The reflectance spectrum is plotted for the different radii of PhC ranges between 120 nm and 170 nm as shown in Figure 2a. As the radius of the PhC varies, we can observe a slight change in the resonant wavelength of the device. The reflectance versus PhC radius is plotted in Figure 2b, which indicates that the lowest reflectance is obtained for the PhC radius = 150 nm, delivering a maximum value of absorbance.

The optimized values of the sensor achieved through best-fitting results are presented in Table 1.

## 3. Methodology

**Figure 3:**
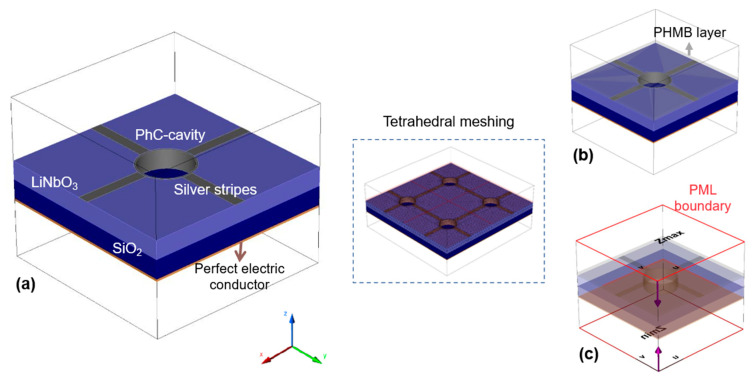
(**a**) Unit structure. (**b**) Unit structure with PHMB functional layer. (**c**) Unit structure signifying source, sink, and PML boundaries of the structure. Moreover, the structure enclosed in the (dotted) square represents the tetrahedral meshing technique for better accuracy in the design process.

The design of the sensor is numerically investigated in CST STUDIO using the frequency domain. Finite-element-method (FEM) is used, which is more efficient in terms of computational power and resources relating to large computational problems [52]. Regarding the accuracy of the design, the tetrahedral meshing technique is used as shown in Figure 3, enclosed by a (dotted) square. Moreover, to save time and computational resources, the unit cell model is used as given in Figure 3a,b. The Floquet periodic boundary conditions (PBC) are used in the (x and y) directions to terminate the simulation domain and open boundaries in the z-direction. Similarly, to absorb the unwanted EM radiations and excitation of higher-energy order modes at the boundary, the perfect matched layers (PML) are used in the open direction, i.e., the z-axis with structure placed in between the source port and the output port. The source port is used to emit the incident EM waves, which in turn are analyzed at the output port. Moreover, within these PML boundaries, two Floquet ports, i.e., Z_max_ and Z_min_, are introduced as shown in Figure 3c, acting as the source and sink to produce the response of the system in terms of reflection and transmission spectra of the EM waves in S-parameters.

To calculate the S-parameters of the transmission and reflection spectra, the ports used for the purpose are *S*_21_ and *S*_11_ and are given by Equations (1) and (2), respectively.

(1) $$S_{21}=\frac{\left(1-Z^{2}\right)\Gamma }{1-Z^{2}\Gamma^{2}}$$

(2) $$S_{11}=\frac{\left(1-\Gamma^{2}\right)Z}{1-Z^{2}\Gamma^{2}}$$

where, *Z* is the impedance parameter, while Γ is the coefficient of the transmission or reflection spectrum depending on the design of the sensor. Moreover, S-parameters, also known as S-matrix or scattering parameter, is used for describing a system in terms of ports in a network or circuit. Here, because the Floquet-boundary ports are used, the S-parameters provides an insight into the relationship between incident EM waves with transmitted or reflected waves at each port. *S*_11_ is the ratio between the reflected wave and the incident wave, whereas *S*_21_ is the ratio between the transmitted and incident wave [53]; hence, all the required parameters can be obtained using these ports analogy. Similarly, the absorbance can also be calculated by Equation (3):

(3) A=1 – T – R

However, the value of the absorbance in this research study depends entirely on the value of reflection as the transmission is approximately zero. Therefore, when the value of the reflectance of the sensor reaches zero, i.e., at the time of the perfect coupling of energy, the value of the absorbance reaches unity at that instant. Similarly, the orientation of the incident source is set in an ‘inward’ direction during the designing process.

## 4. Results

The performance of the sensing device in the presence of different concentrations of the CO_2_ gas in terms of change in the refractive index of the PHMB functional layer is determined; thus, the values of the refractive index determining these different concentrations of the CO_2_ gas used are referenced from [34], and given in Table 2.

**Figure 4:**
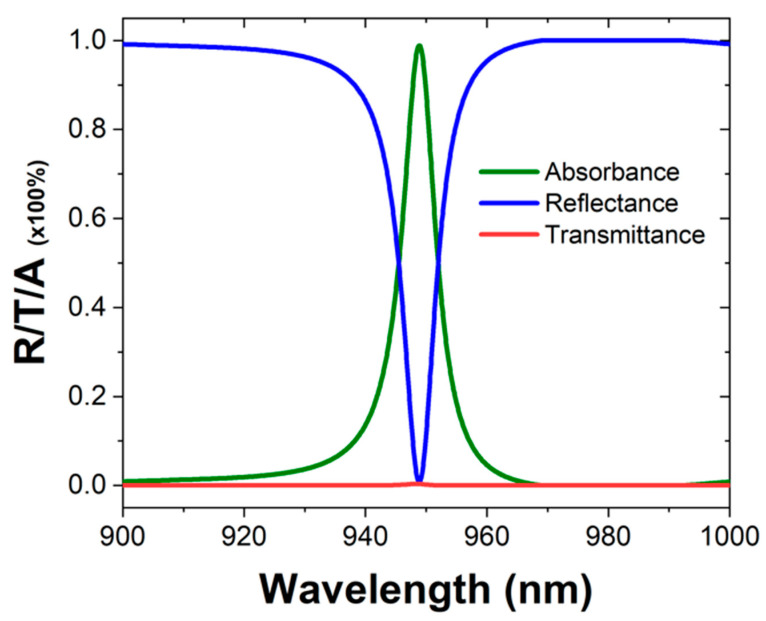
R/T/A output spectra of the CO_2_ gas sensor.

To observe the spectral properties of the designed sensor, the reflectance/transmittance/absorbance *(R/T/A)* spectra of the sensor are shown in Figure 4. The spectra show zero transmission and high absorbance at the resonant wavelength. The resonant oscillations in the perfect absorber can be tailored to match the frequency and polarization of the incoming radiation, leading to a highly efficient absorption of the radiation. This is achieved by tuning the geometrical parameters of the resonant elements, such as their size, shape, and spacing, to achieve a specific resonant response.

**Figure 5:**
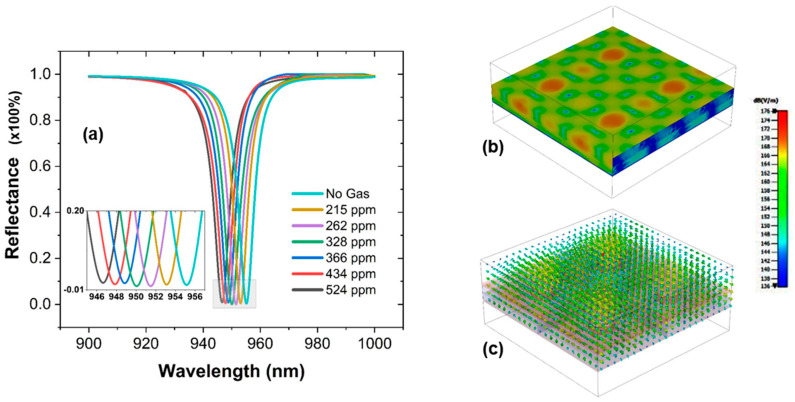
(**a**) Output reflection spectra, presenting a blue shift in the resonant wavelength λ_res_ of the sensor sensing different concentrations of the CO_2_ gas. (**b**) Plasmonics effects on the surface of the sensor. (**c**) Energy confinement by the sensor.

Therefore, after depositing a PHMB layer on the surface of the device, the value of the refractive index is varied and presented against different concentrations of the CO_2_ gas, as shown in Figure 5a, investigating a blue shift in terms of the resonant wavelength λ_res_ of the sensor, i.e., 955–946 nm. This is due to the fact, that initially, when there is no ambient CO_2_ gas, the value of the refractive index of the PHMB layer is 1.55 and the distribution of molecules within the PHMB layer is uniform and homogenous. However, after increasing the concentration of the CO_2_ gas, i.e., 0 to 524 ppm, the value of refractive index of the PHMB layer tends to decrease, i.e., 1.55–1.48, as molecules of the CO_2_ starts binding with the molecules of PHMB layer that in turn causes an increase in variation in the distribution formation of the previously homogenous molecules into a more heterogenous cluster within the layer. This effect has a direct impact on the refractive index of the monitoring layer. Figure 5b,c investigate the plasmonic effects and confinement of energy by the sensor, respectively.

In all types of sensors, sensitivity is a vital aspect that is considered imperative in the design phase. It is also considered a key element to verify the performance and working of a sensor according to the provided conditions. The sensitivity is defined as the ratio of change in the resonant wavelength $\delta \lambda_{res}$ to that of change in the concentration of the CO_2_ gas (δconc.), as given in Equation (4)

(4) $$S=\frac{\delta \lambda_{res}\;}{\delta conc.}$$

**Figure 6:**
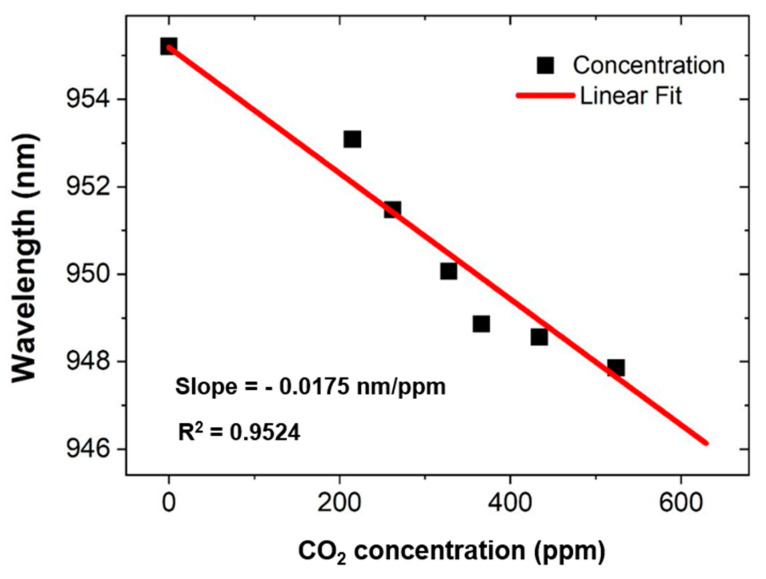
Linear response of the sensor for different concentrations of the CO_2_ gas.

Furthermore, the sensor presents a linear correlation for 0–524 ppm of the concentration of the CO_2_ gas as shown in Figure 6, which is desirable for the measurement of the atmospheric CO_2_ gas. Considering the performance of the sensor, it achieves the highest, i.e., 17.32 pm/ppm, and lowest, i.e., 9.5 pm/ppm, values of sensitivity for different concentration values of the CO_2_ gas. It is important to note that the relationship of sensitivity of the sensor with that of the concentrations of CO_2_ gas is not linear, but rather, it increases gradually up to 366 ppm of a concentration of the CO_2_ gas and provides the highest value at 366 ppm; however, later on it plummets very slightly, as shown in Table 3. This effect is not fully understood by the authors, but considered normal according to certain design or material factors, albeit the overall sensitivity of the sensor remains considerably higher for the remaining concentration sample values of the CO_2_ gas, i.e., 366–524 ppm, which increases the significance of the designed sensor. Table 3 exhibits a comprehensive performance of the sensor in terms of sensitivity and change in the resonance wavelength δλ_res_.

Figure of merit (FOM) is another parameter used to verify the device’s suitability and reliance on a specific task. It is a quantitative measure of a sensor’s overall performance, usually expressed as a single value that combines several important parameters. FOM can help in comparing different sensors and selecting the most appropriate one for a particular application. It is defined as the ratio of the sensitivity of a sensor to the full width at half maximum (FWHM) of the peak value, which is provided by Equation (5) as:

(5) $$Figure\;of\;Merit\;\left(FOM\right)=\frac{S}{FWHM}$$

**Figure 7:**
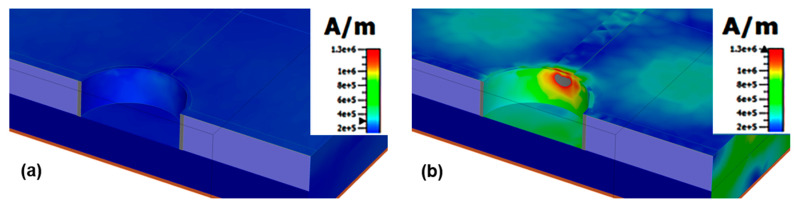
(**a**) H-field mapping for λ_res_ = 900 nm, (**b**) H-field distribution for λ_res_ = 949 nm.

The FWHM of the proposed sensor is measured as 6.0 nm with the bulk sensitivity at 17.32 pm/ppm. As a result, the FOM value achieved by the sensor according to Equation (5) is 2.90 RIU^−1^. Figure 7a,b present the H-field distribution for λ = 900 nm and λ = 949 nm, respectively. It can be seen that at the non-resonant wavelength (Figure 7a), no EM energy is confined in the cavity, whereas at the resonant wavelength (Figure 7b), maximum field power is confined in the cavity, resulting in the maximum absorption of light at resonant wavelength, using the basic modes of polarization.

A comparative study of the designed sensor with previously investigated structures is given in Table 4. The sensors that are close to the design and sensitivity of this research are selected in terms of designing layers, compactness, and overall sensitivity.

## 5. Suggested Fabrication Steps

**Figure 8:**
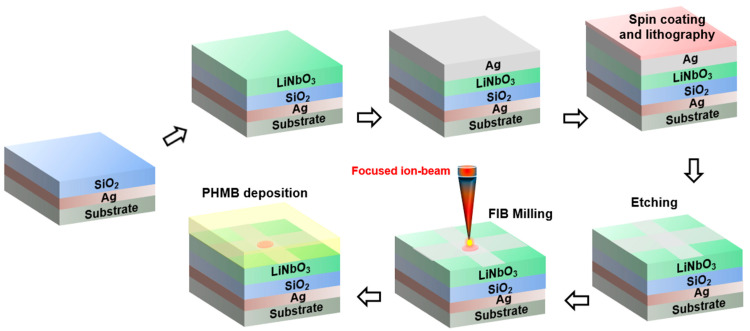
The stepwise proposed fabrication of the CO_2_ gas sensor using the host functional layer.

The complete fabrication process of the structure is shown in Figure 8. Mainly, the fabrication steps involve the deposition of thin films, the lithography process, and etching. In the first step, the layer of PEC, i.e., Ag, can be deposited on a glass substrate to form the base of the design using electron beam-assisted thermal evaporation [54], followed by a layer of SiO_2_, which can be deposited using plasma-enhanced chemical vapor deposition (PECVD) or ion-beam sputter deposition (IBSD) [55]. In the third step, a layer of LiNbO_3_ can be deposited via the procedure of pulse laser deposition (PLD), providing better accuracy and control over the deposition process [56]. For the implementation of the PhC-cavity, focused ion-beam (FIB) technology can be used. Similarly, the Ag stripes can be deposited using the focused electron-ion beam-induced deposition (FEBID) technique [57]. After completion of the structural layout of the sensor, the functional layer of PHMB can be deposited using spin coating [58]. Moreover, by controlling the speed of the spin coating and solution concentration, the desired thickness of the PHMB layer can be achieved.

## 6. Conclusions

A plasmonics-based perfect absorber CO_2_ gas sensor using LiNbO_3_ is investigated in this research study. The sensor comprises PhC-cavity, Ag stripes, and a host-functional material, i.e., polyhexamethylene biguanide polymer (PHMB), for sensing CO_2_ gas. The LiNbO_3_ layer is used to enhance EM fields, with PhC-cavity implemented within the middle of the cell to enable penetration of the EM waves further into the sublayers and Ag stripes for the plasmonics effects. Therefore, on sensing the CO_2_ gas, the refractive index of the host material decreases due to the fact that, as the CO_2_ gas molecules are absorbed by the functional material, it causes variation in the distribution of the molecules/electrons of the functional layer. As a result, its refractive index decreases, producing a blue shift in the resonant wavelength in its reflection spectra. The sensor thus provides a sensing capability to different concentrations of the CO_2_ gas in the range, i.e., 0–524 ppm, with a maximum sensitivity of −17.23 pm/ppm achieved for 366 ppm of the concentration with a FOM value of 2.9 RIU^−1^. Therefore, the concluded approach presents a simple and compact design of the sensor. In relation to the sensor performance and sensitivity, it can be used in several fields for the detection of gases using suitable host-functional material.

## Figures and Tables

**Table 1:** Optimized structural values of the CO_2_ sensor.

Layers (Bottom to Top)	Materials	Values
Layer 1	PEC (Ag)	10 nm
Layer 2	SiO_2_	100 nm
Layer 3	LiNbO_3_	100 nm
PhC-cavity	Air	150 nm (radius)
Layer 4	Ag stripes	1 nm (height), 60 nm (width)
Layer 5	Functional layer (PHMB)	30 nm

**Table 2:** Refractive index values of the PHMB functional layer for different concentrations of the CO_2_ gas.

Refractive Index (n)	CO_2_ Gas Concentration
1.55	0
1.54	215
1.53	262
1.52	328
1.51	366
1.49	434
1.48	524

**Table 3:** Detailed performance of the sensor using different concentrations of the CO_2_ gas.

Application Area	Refractive Index (n)	Concentration CO_2_ (ppm)	λ_res_ (pm)	δλ_res_ (pm)	Sensitivity (pm/ppm)
**CO_2_ gas sensing**	1.55	0	955,210	-	-
	1.54	215	953,170	2040	9.5
	1.53	262	951,480	3730	14.23
	1.52	328	950,170	5040	15.36
	1.51	366	948,870	6340	17.32
	1.49	434	947,870	7340	16.91
	1.48	524	946,670	8540	16.29

**Table 4:** Comparative study of the research with previously investigated studies.

Sensor Designs	Sensitivity (pm/ppm)	Research Work
Quartz layer + Au layer + nano atoms + functional layer	17.30	[34]
Fabry–Perot cavity + single mode fiber + functional layer	12.20	[37]
Micro-bubble + tapered fiber + functional layer	0.46	[39]
LiNbO_3_ layer + silver stripes + nano-cavity + functional layer	17.32	***This work***

## Data Availability

Not applicable.
